# Acute Intestinal Invagination: An Exceptional Method of Revealing Crohn’s Disease

**DOI:** 10.7759/cureus.18673

**Published:** 2021-10-11

**Authors:** Rachid Jabi, Siham Elmir, Mouad Ouryemchi, Mohammed Bouziane

**Affiliations:** 1 Department of General Surgery, Faculty of Medicine and Pharmacy, Mohamed Vi University Hospital, Oujda, MAR; 2 Laboratory of Anatomy, Microsurgery and Surgery Experimental and Medical Simulation (LAMCESM), Mohammed Ist University, Oujda, MAR; 3 Department of Physical Medicine and Rehabilitation, Faculty of Medicine and Pharmacy, Mohammed VI University Hospital, Oujda, MAR; 4 Department of General Surgery, Faculty of Medicine and Pharmacy, Mohammed VI University Hospital, Oujda, MAR

**Keywords:** gastrointestinal manifestation, management, surgery, crohn's disease, invagination

## Abstract

Crohn's disease presents a public health problem. The clinical presentation is variable with gastrointestinal and extra gastrointestinal manifestations. The management is multidisciplinary while patients with Crohn's disease rarely require surgery. We present a rare case of a 57-year-old patient followed for 10 years for ankylosing spondylitis and admitted for abdominal pain on intussusception caused due to Crohn's disease which was probably symptomatic but not understood by your patient.

## Introduction

Crohn's disease is defined as an idiopathic and chronic intestinal inflammation with an incidence exceeding 0.3% [1]. Its presentation is variable and its management is multidisciplinary [2]. We report following the SCARE recommendations [3] a sporadic case of Crohn's disease discovered following intussusception as an exceptional revealing mode. We hope through this very rare case to enrich the poor literature published on this subject and underline the place of group discussion before the discovery of this chronic pathology.

## Case presentation

**Figure 1:**
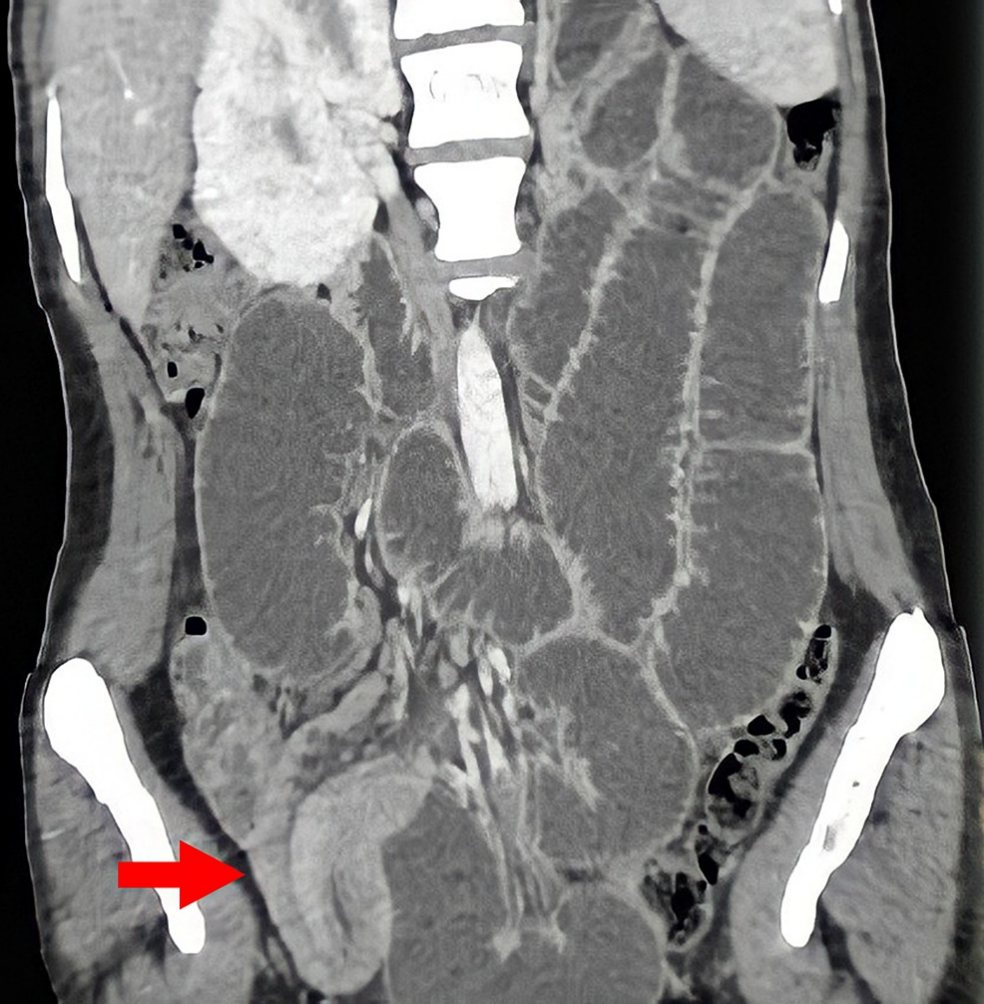
CT scan showing ileo-ileal invagination producing a sandwich image (red arrow)

This is a 57-year-old male patient from eastern Morocco, followed up for 10 years for ankylosing spondylitis and admitted to the emergency room for an occlusive syndrome and abdominal pain. The symptomatology goes back two years with the onset of intermittent postprandial abdominal pain in the right iliac fossa which worsened on the day of his admission to the hospital. The clinical examination of our patient revealed tenderness in the right iliac fossa with palpation of a mobile mass measuring about 4 cm and free hernial orifices. The standard biological assessment carried out in the emergency room had objectified an inflammatory syndrome with a CRP increased to 60 mg/L and the CT scan had objectified a cockade image next to the last ileal handle. This image, which is probably related to an ileo-ileal invagination, is also the site of a circumferential thickening, regular symmetrical, measuring 14 mm in maximum thickness with respect for the adjacent fat and without individualization of peri-lesional lymphadenopathy (Figure 1).

**Figure 2:**
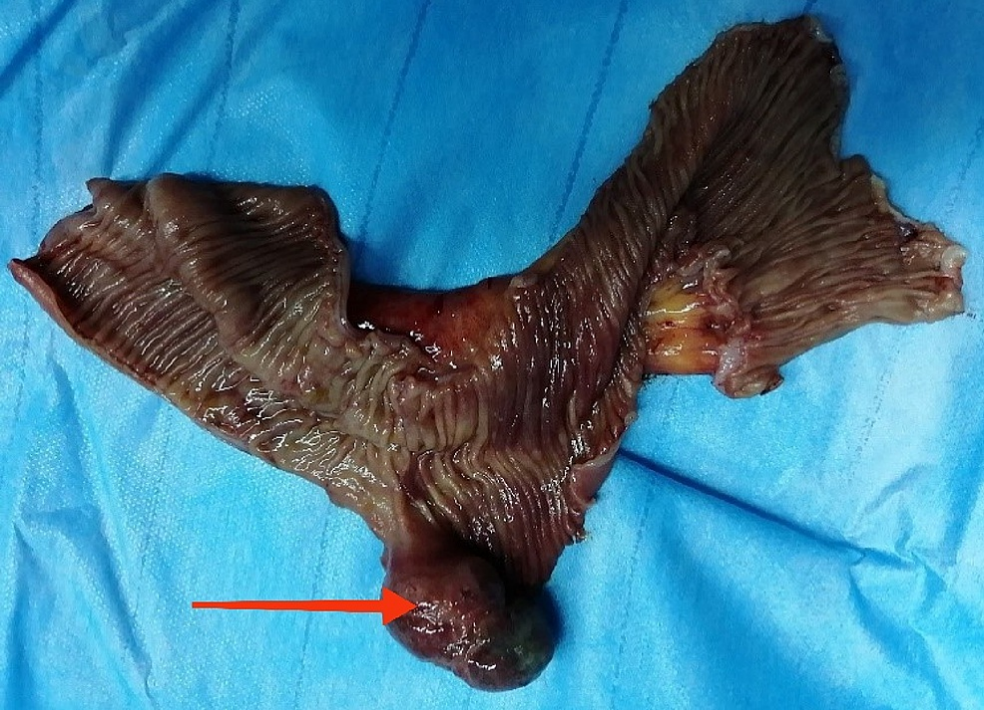
Open surgical specimen showing intussusception on the polyp (Crohn's disease)

**Figure 3:**
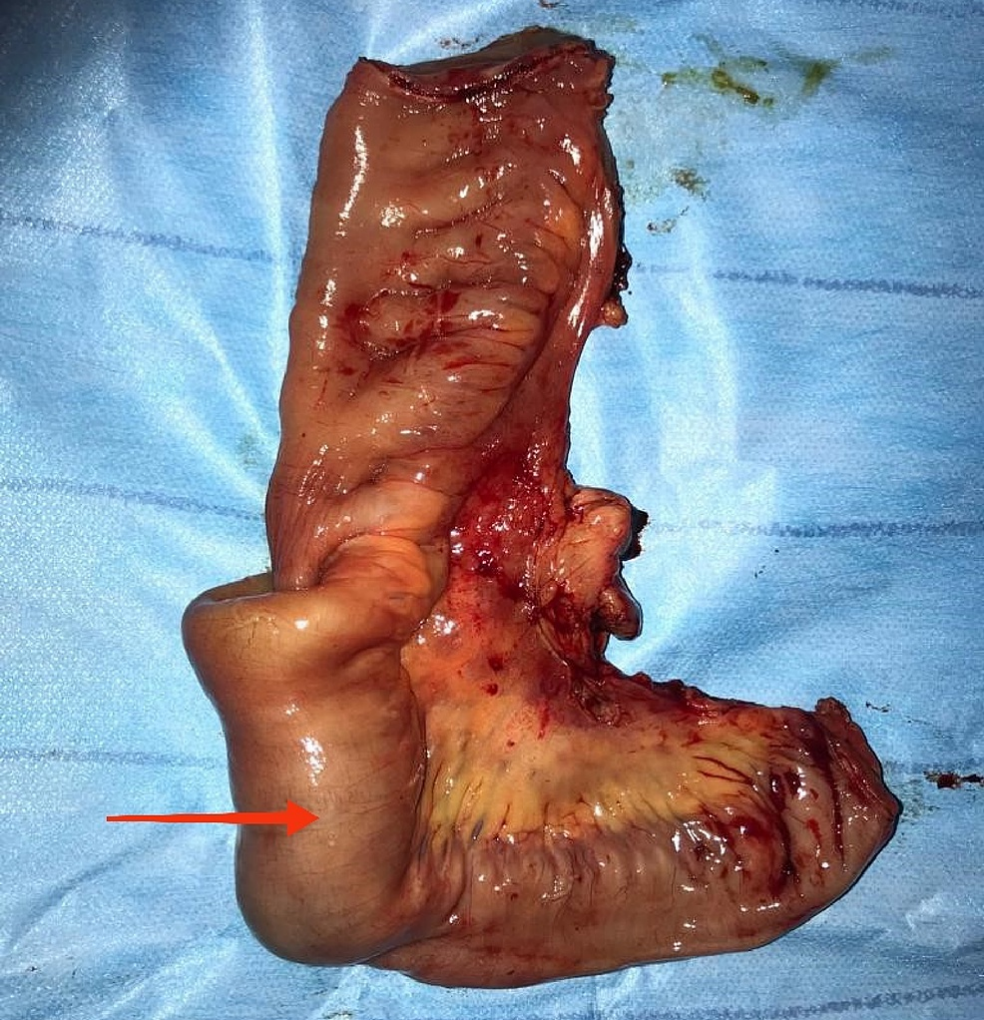
Operative segmental resection piece showing intussusception

After multidisciplinary discussion and discussion with the patient, we opted for surgical exploration performed under general anesthesia which had demonstrated an intussusception 50 cm from the last intestinal loop on an intraluminal process with mesenteric lymphadenopathy. Bowel resection with lymphadenectomy involving intussusception with manual anastomosis was performed (Figures 2, 3).

**Figure 4:**
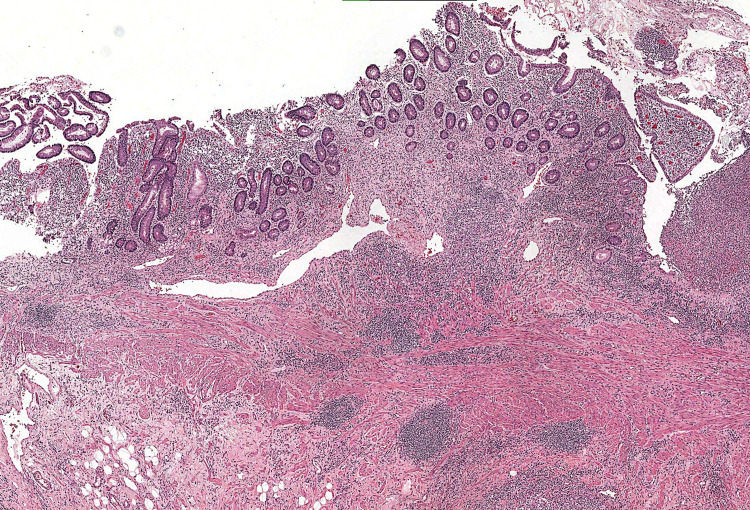
Microphotography showing an ileal mucosa with numerous anomalies, suggesting Crohn's disease: pyloric gland metaplasia, dense inflammatory infiltrate with numerous cryptic abscesses and the presence of mucosal fissure (left of the field) (HE, 100x)

**Figure 5:**
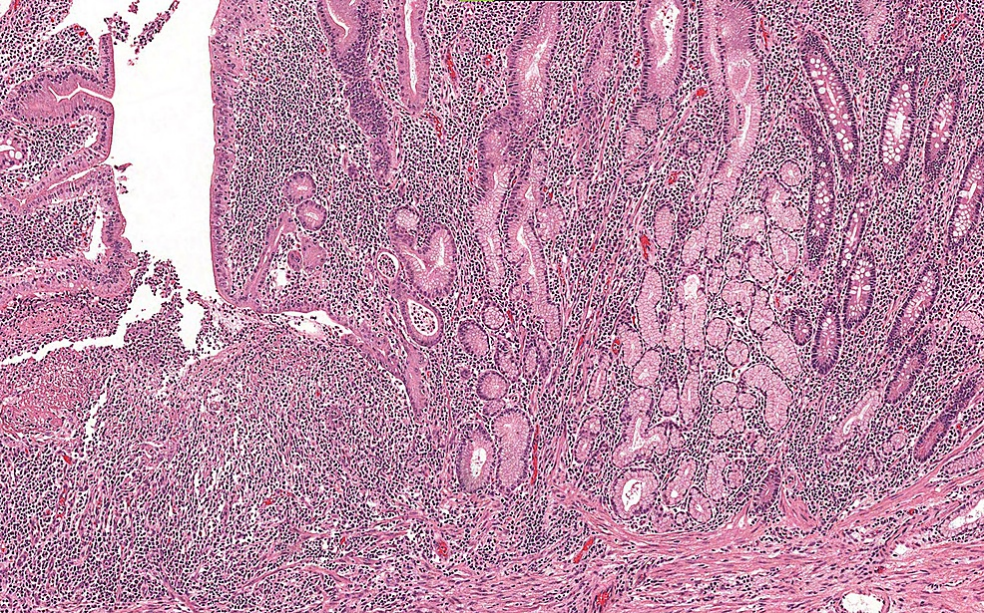
Microphotography of a polypoid formation from the same resected specimen, showing its inflammatory nature with ulcerations in either side of the polyp (HE, 40x)

The pathological study noted the presence of an inflammatory pseudo-polyp integrating into chronic inflammatory bowel disease Crohn's type (Figures 4, 5). The patient's postoperative progress course was uneventful, our patient was satisfied with the overall care and the discharge was carried out for five days under specific medical treatment for Crohn's disease. Follow-up after two years was without abnormalities with control of rheumatological and digestive manifestations.

## Discussion

Crohn's disease is defined by chronic and idiopathic intestinal inflammation; it affects ulcerative colitis about 1.5 million inhabitants in the USA [1]. Its diagnosis is based on a clinical picture supported with radiological and histological findings [2], which makes this pathology a polymorphic entity [4]. The clinical presentation varies from intestinal to extra-intestinal manifestations [5], which can be revealing and even precede digestive signs for several years like our case which was followed for ankylosing spondylitis before retaining the diagnosis of Crohn's disease [6].

The management of this chronic disease is subject to the guidelines of several consensuses and conferences [7] in which medical treatment is an obligatory step throughout the treatment [8]. Surgery, which is one of the therapeutic means of this pathology, is subject to several constraints such as the preservation of the digestive tract by sequelae, its indications must follow the recommendations of scientific societies [9]. This surgery can be scheduled according to certain criteria [10] or in emergencies [11] and sometimes it is indicative of this disease as was the case with our patient. The discovery of a sequel Crohn was reported in the form of rare case reports of ileo-ileal intussusception in the literature [12] and in the form of a sporadic case of intussusception on a fibrinoid polyp revealing this disease as presented in our case. The management of intussusception in adults is subject to several recommendations [13,14] and should take into consideration secondary etiologies such as tuberculosis [15], lipoma [16], cancer [17], and rarely Crohn's disease [12]. Consequently, the file was discussed in multidisciplinary staff, and the decision to explore the patient under laparoscopy was retained as recommended.

Limitations

Our report is summed up in the presentation of a single rare and sporadic case for which the management is not codified, thus offering us the drafting of another report integrating all the cases published through the literature with a large sampling allowing us to unify the therapeutic management.

## Conclusions

The particularity of our patient lies in the rarity of the clinical form and that it is preceded several years by extra gastrointestinal manifestations of this disease. We insist on multidisciplinary management of system pathologies such as ankylosing spondylitis on digestive endoscopic exploration and the use of CT imaging for any unusual signs.
